# Diagnostic Accuracy of Body Mass Index in Defining Childhood Obesity: Analysis of Cross-Sectional Data from Ghanaian Children

**DOI:** 10.3390/ijerph17010036

**Published:** 2019-12-19

**Authors:** Theodosia Adom, André Pascal Kengne, Anniza De Villiers, Rose Boatin, Thandi Puoane

**Affiliations:** 1 School of Public Health, Faculty of Community and Health Sciences, University of Western Cape, Cape Town 7535, South Africa; tpuoane@uwc.ac.za; 2 Nutrition Research Centre, Radiological and Medical Sciences Research Institute, Ghana Atomic Energy Commission, Accra LG80, Ghana; roseboatin@yahoo.com; 3 Non-Communicable Disease Research Unit, South African Medical Research Council, Cape Town 7505, South Africa; Andre.Kengne@mrc.ac.za; 4 Division of Research Capacity Development, South African Medical Research Council, Cape Town 7505, South Africa; Anniza.DeVilliers@mrc.ac.za

**Keywords:** body mass index, obesity, deuterium oxide, percent body fat, sensitivity, specificity, accuracy, school children

## Abstract

Background: Screening methods for childhood obesity are based largely on the published body mass index (BMI) criteria. Nonetheless, their accuracy in African children is largely unknown. The diagnostic accuracies of the World Health Organization (WHO), the Centers for Disease Control and Prevention (CDC), and the International Obesity Taskforce (IOTF) BMI-based criteria in defining obesity using deuterium dilution as a criterion method in a sample of Ghanaian children are presented. Methods: Data on anthropometric indices and percent body fat were collected from 183 children aged 8–11 years. The sensitivity, specificity, and predictive values were calculated. The overall performance of the BMI criteria was evaluated using the receiver operating characteristics area under the curve (AUC). Results: Overall sensitivity of WHO, CDC, and IOTF were 59.4% (40.6–76.3), 53.1% (34.7–70.9), and 46.9% (29.1–65.3) respectively. The overall specificity was high, ranging from 98.7% by WHO to 100.0% by IOTF. The AUC were 0.936 (0.865–1.000), 0.924 (0.852–0.995), and 0.945 (0.879–1.000) by the WHO, CDC, and IOTF criteria respectively for the overall sample. Prevalence of obesity by the WHO, CDC, IOTF, and deuterium oxide-derived percent body fat were 11.5%, 10.4%, 8.2%, and 17.5% respectively, with significant positive correlations between the BMI z-scores and percent body fat. Conclusions: The BMI-based criteria were largely specific but with moderate sensitivity in detecting excess body fat in Ghanaian children. To improve diagnostic accuracy, direct measurement of body fat and other health risk factors should be considered in addition to BMI.

## 1. Introduction

Obesity is a major risk factor for non-communicable diseases [1]. Among children, obesity may present significant health and psychological problems including type 2 diabetes, increased risk of developing hypertension, high cholesterol, orthopedic problems, and low self-esteem [1,2]. The adverse health consequences associated with obesity are related to excess body fat, calling for accurate methods for diagnosis, particularly among children. At present, the screening methods for obesity are based largely on the body mass index (BMI), calculated as body weight (in kg) divided by the square of the height (in meters). Despite its inherent limitations to distinguish between fat mass (FM) and fat-free mass (FFM), both of which contribute to the BMI [3], BMI has been traditionally used in epidemiological studies as a proxy for adiposity because of its relative simplicity and affordability. Nonetheless, BMI is a measure of excess weight rather that excess body fat and changes with age, gender, and maturation in children [3]. 

Different BMI criteria have been developed for the classification of weight status. These are: The World Health Organization (WHO) reference [4], derived from the z-score of the mean BMI-for-age after computing standard deviations; the Centers for Disease Control and Prevention (CDC) [5], based on the BMI-for-age percentile methodology; and the International Obesity Taskforce (IOTF) [6] definition from the lambda, mu, and sigma (LMS) methodology for the calculation of the z-score. 

A number of reference methods are available to measure body fatness including under water weighing, dual-energy X-ray absorptiometry (DXA), total body potassium, air displacement plethysmography, bioelectrical impedance, and isotope dilution method [7]. Some of these methods are limited to laboratory settings, costly, and may not be suitable for children [7,8]. The isotope dilution method is one of the safe, non-invasive methods for body composition assessment that enables measurements of body fat under free-living conditions [8]. 

Most studies among children and adolescents in Africa apply BMI-based criteria to estimate overweight and obesity [9]. Nonetheless, at the continental level, the diagnostic accuracy of the published BMI references to detect excess body fat among children is largely unknown; only few studies have compared the BMI criteria against a criterion measure of body fat [10,11]. The present study aims to evaluate the diagnostic accuracies of the three international BMI the CDC, IOTF, and WHO based criteria in defining obesity using deuterium dilution as a criterion method in a sample of Ghanaian primary school children.

## 2. Materials and Methods 

This is a cross-sectional analysis of 8–11-year-old school children in six primary schools in an urban area in Ghana. Data on anthropometric indices and percent body fat were collected. 

### 2.1. Study Population

A convenient sample of 183 children from three private and three public schools in the Adentan Municipality of the Greater Region of Ghana were randomly selected. The participants were a sub-sample from a larger study on childhood obesity involving 543 children from 14 schools comprising 111 girls and 72 boys. Details of the recruitment and selection of participants have been previously described [12]. 

### 2.2. Data Collection

#### 2.2.1. Anthropometry

Body weight was measured to the nearest 0.1 kg with a digital scale (Seca 869, GmbH & Co., Hamburg, Germany). Children were weighed in their school uniforms but asked to remove shoes, socks, watches, sweaters, jackets, and items in the pockets. Height was measured to the nearest 0.1 cm using the Shorr Board (Shorr Productions, Olney, MD). All measurements were done in duplicates. The means of the duplicate measurements were used to compute BMI as body weight (in kilogramme) divided by height (in meters)^2^. BMI-for-age was calculated and obesity defined as BMI-for-age z-score ≥ +2.0 SD by WHO [4]; BMI-for-age percentile ≥ 95th percentile by CDC [5]; and BMI-for-age z-score ≥ 30 kg/m^2^ by IOTF adjusted to reflect the cut-off point at age 18 years [6].

#### 2.2.2. Total Body Water for Percent Body Fat Estimation

Body fat of participants was assessed using the deuterium dilution method with Fourier transform infrared spectrometer (FTIR) following the International Atomic Energy Agency (IAEA) guidelines [13]. Children were asked not to eat for at least two hours prior to data collection. Pre-dose saliva samples were collected after which each child received a dose of given weight of deuterium oxide labelled water (99.8% purity, Cambridge Isotope Laboratories Inc. Andover, MA) based on their body weight [13] to drink. The doses were prepared in batches prior to the study. No food or drinks were allowed during equilibration period. Two additional samples were collected 3 and 3.5 h after drinking the dose. The samples were transported on ice to the laboratory and stored at −20 °C prior to analysis. 

Deuterium enrichment of the saliva samples were measured using FTIR (Shimadzu IRPrestige-21, Vienna, Austria) at the Ghana Atomic Energy Commission following IAEA guidelines [13]. Samples were analyzed in duplicates and the mean enrichment for each time point was calculated. Total body water (TBW) was calculated from the mean deuterium enrichment at time zero with the use of a correction factor (deuterium space) for non-aqueous dilution of deuterium oxide using the age-and gender- specific values for the hydration of FFM for children [14]. Where only one time point data was available, that was used to calculate the TBW.

Deuterium space = dose ingested (mg)/deuterium enrichment of saliva (mg/kg) 

TBW (kg) = deuterium space/1.041

FFM was computed from the TBW as: 

FFM (kg) = TBW (kg)/age-and gender-specific hydration factor 

The FM was calculated as the difference between body weight and the FFM and expressed as a percentage of the body weight. 

Fat mass = Weight (kg) − FFM (kg); expressed as percentage

There is no universally accepted definition for excess body fat using isotope dilution methods in children. For the purposes of this analysis, excess body fat was defined as body fat >25% for boys and >30% for girls, respectively [15]. 

#### 2.2.3. Ethical Considerations

Ethics approvals were obtained from the Senate Research Committee of the University of Western Cape (ID NO: 15/5/5) and Ethical Review Committee of the Ghana Health Service (ID NO: GHS-ERC: 01/07/13). Approval was also obtained from the Municipal Education Directorate of the Ghana Education Service and from the heads of participating schools. Since the study involved children under 16 years, written informed consent were obtained from the parents or legal guardians of the children. Verbal assent was given by each participating child after explaining the study. 

#### 2.2.4. Statistical Analysis

All analyses were performed with Stata 13.0 (StataCorp). Results are expressed as means (±SDs) or medians (and 25th–75th percentiles) for continuous variables, while categorical variables are reported as frequencies and percentages. Chi-square test, Student t-test, and the Mann–Whitney U test were used for comparison as appropriate. A Spearman’s correlation was used to assess the relationship between percent body fat and WHO, CDC, and IOTF BMI-for-age z-scores. The performance of the published BMI criteria to discriminate children with excess body fat from those with normal body fat (childhood obesity) was evaluated. The sensitivity (the proportion of children with excess body fat who have high BMI-for-age z-scores), specificity (the proportion of children who do not have excess body fat and who do not have high BMI-for-age z-scores), positive predictive value (proportion of children with high BMI-for-age z-scores who have excess body fat), negative predictive value (proportion of children with low BMI-for-age z-scores who do have excess body fat), and receiver operating characteristics (ROC) curves and area under the curves for the BMI references, were computed. Statistical significance was set at *p* < 0.05.

## 3. Results

### 3.1. Descriptive Characteristics and Obesity Prevalence of Children

The descriptive characteristics and prevalence of obesity by the different diagnostic criteria are summarized in Table 1. The median (25th–75th percentiles) age of the study participants was 10 (9–10) years. No significant difference was observed between boys and girls in weight, height, and BMI. However, the girls had significantly higher percent body fat compared to the boys (21.3% vs. 14.7%; *p* < 0.0001). 

**Figure 1:**
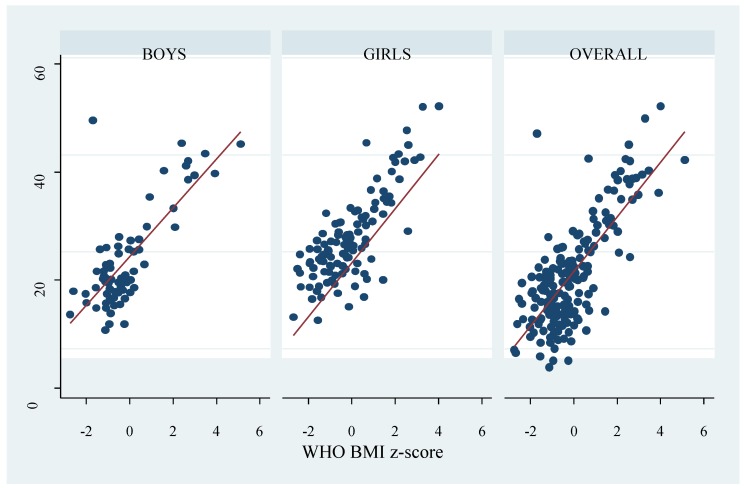
Correlation between percent body fat measured by the deuterium dilution method and World Health Organization (WHO) body mass index (BMI) z-score. The dots represent percent body fat and the red line represent the fitted values.

**Figure 2:**
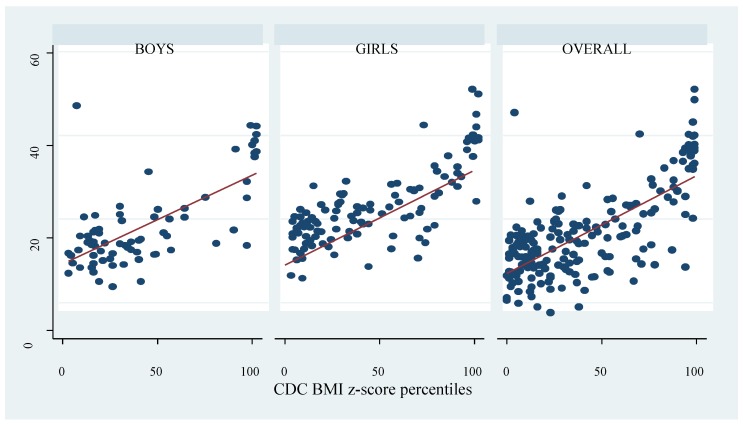
Correlation between percent body fat measured by the deuterium dilution method and Centers for Disease Control and Prevention (CDC) BMI z-score percentiles. The dots represent percent body fat and the red line represent the fitted values.

**Figure 3:**
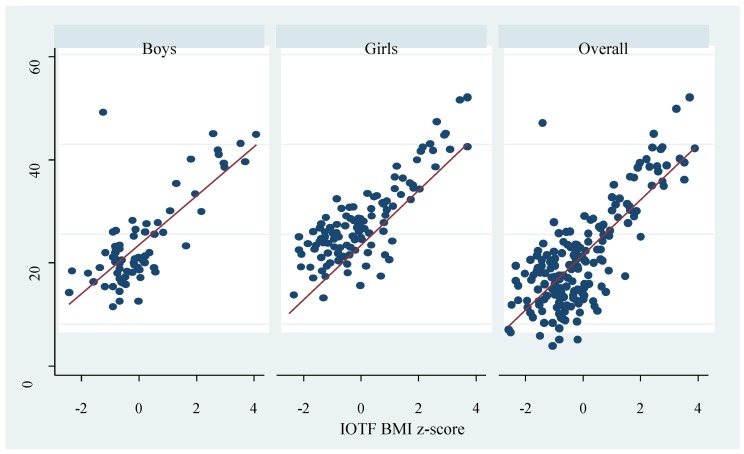
Correlation between percent body fat measured by the deuterium dilution method and International Obesity Taskforce (IOTF) BMI z-score. The dots represent percent body fat and the red line represent the fitted values.

Obesity prevalence appears to vary by gender and the criteria used; nonetheless, the differences were not significant. The overall prevalence based on the WHO, CDC, IOTF, and percent body fat determined by the deuterium method were 11.5%, 10.4%, 8.2%, and 17.5% respectively. Across criteria, the overall highest obesity prevalence was by percent body fat measured with the deuterium dilution method; 18.0% girls and 16.7% boys were classified as obese (*p* = 0.814). Prevalence based on the WHO criteria was 13.9% among boys and 9.9% among girls (*p* = 0.409). Using the CDC and IOTF cut-offs, the prevalence among boys was 11.1% and 8.3%, and 9.0% and 8.1% among girls respectively. Except for the deuterium method, all diagnostic criteria classified higher proportion of boys as obese compared to girls, although the differences were not significant. There were significant positive correlations between the BMI z-scores and percent body fat (Figure 1, Figure 2 and Figure 3). Across criteria, the correlation coefficient rho (ρ) was 0.638, *p* < 0.0001, 0.635, *p* < 0.001, and 0.625, *p* < 0.0001 for WHO, CDC, and IOTF, respectively. By gender, the corresponding values for WHO, CDC, and IOTF were higher in girls (0.694, 0.713, 0.719, all *p* < 0.0001) compared to boys (0.550, 0.532, and 0.562, all *p* < 0.0001).

### 3.2. Diagnostic Accuracy and Performance of BMI Criteria for Defining Obesity

The diagnostic performance of BMI-based criteria for classifying deuterium method-based obesity is presented in Table 2. The overall sensitivity was 59.4% (40.6–76.3), 53.1% (34.7–70.9), and 46.9% (29.1–65.3) by WHO, CDC, and IOTF criteria, respectively. The sensitivity was high among boys with the WHO criterion 75.0% (42.8–94.5) and low among girls using the CDC and IOTF criteria, 45.0% (23.1–68.5). By contrast, the specificity was high across the criteria in the overall sample ranging from 98.7% (95.3–99.8) by WHO to 100.0 (97.6–100.0) by IOTF. Further, the positive predictive values (PPV) in the overall sample ranged from 90.5% (69.6–98.8) by WHO to 100.0% (80.5–100.0) by IOTF. The negative predictive values (NPV) were higher with WHO-based criterion; 91.9% (86.7–95.7) and 89.9% (84.3–94.0) by IOTF. The sensitivity, specificity, PPV, and NPV were similar across all criteria by gender.

**Figure 4:**
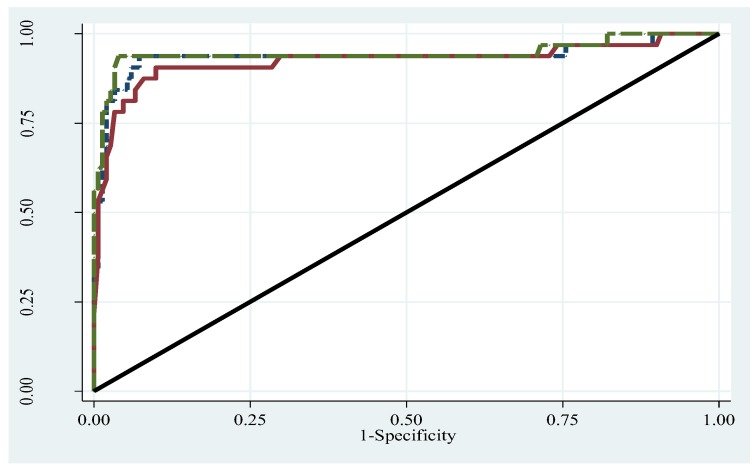
Receiver operating characteristics curve for WHO, CDC, and IOTF criteria. Long broken forest green line: ROC area for IOTF, AUC = 0.945. Dash navy line: ROC area for WHO, AUC = 0.936. Solid maroon line: ROC area for CDC, AUC = 0.924. Diagonal line: ROC area for reference, AUC = 0.500.

The overall accuracy and performance analysis of the BMI-for-age z-score and BMI-for-age percentiles in identifying obese children is indicated by the receiver operating characteristics (ROC) area under the curve (AUC). The AUC areas were 0.936 (0.865–1.000), 0.924 (0.852–0.995), and 0.945 (0.879–1.000) by the WHO, CDC, and IOTF criteria, respectively, for the overall sample (Figure 4). The AUC did not differ in the overall sample and by gender (Supplementary Figures S1–S3) for all criteria.

### 3.3. Empirical Cut-Point Estimation for Defining Obesity

Using the Youden index J point approach of the empirical cut-point estimation (Table 3), the WHO, CDC, and IOTF cut-points that optimize sensitivity, specificity, PPV, and NPV for obesity were 0.68, 69.5%, and 0.50 respectively for the overall sample. The corresponding optimal cut-offs by gender were 0.86, 87.5%, and 0.50 for boys; and 0.68, 69.5%, and 0.50 for girls.

## 4. Discussion

This study provides the findings of the accuracy of the published BMI z-score for WHO, CDC, and IOTF to detect excess body fat in pre-adolescent school children in sub-Saharan Africa. The results show that BMI as an indicator of obesity had high specificity with mostly high predictive values across diagnostic criteria. Nonetheless, none of the published criteria achieved optimal rates of sensitivity. Across criteria, at least 40% of the children who were obese were misclassified. Area under the ROC curve indicated that BMI is an acceptable tool for diagnosing excess body fat. Moreover, the diagnostic accuracy of the WHO, CDC, and IOTF references were similar across the overall samples and also when stratified by gender. We observed positive correlations between the deuterium-derived percent body fat and the published BMI z-scores. Furthermore, the optimal BMI cut-off points for defining obesity, as determined for the present sample were lower; 0.86 for boys, 0.68 for both girls, and overall by WHO reference; 87.5% for boys, 69.5% for both girls, and overall samples by CDC reference; and 0.50 for boys, girls, and overall sample by IOTF reference. 

Several factors are known to influence the diagnostic performance of BMI-based criteria to detect excess body fat. These include the methods of body composition assessment, the cut-offs to define excess percent body fat in the evaluation of the BMI-criteria, and characteristics of the reference population such as ethnicity, maturity, and gender [16,17,18,19,20]. The low-to-moderate sensitivity and high specificity reported in the present study are generally consistent with the literature [11,17,21,22,23,24]. Furthermore, BMI underestimated adiposity in South Asian children while among children of African origin, body fat was overestimated [18,19]. The present results are contrasting to the findings from a systematic review and meta-analysis of the diagnostic performance of BMI, where a pooled sensitivity of 73.0% and specificity of 93.0% were reported [25]. 

Although the methodologies for assessing body fat may differ, many studies have consistently reported low sensitivity of IOTF criterion compared with other criteria for diagnosing childhood obesity [17,23,26,27]. For example, using multisite skinfold thicknesses as the measure of body fat, Zimmermann et al. [23] found that IOTF criterion had low sensitivity relative to the CDC in a national sample of 6–12 year old Swiss children. Deurenberg-Yap et al. [17] also observed lower overall prevalence of obesity by IOTF criterion compared to CDC criterion in Asian adolescents. In another study, BMI percentiles had low sensitivity but high specificity in Italian school children aged 8–12 years [22]. On the other hand, BMI showed higher sensitivities and moderate specificities in Brazilian children aged 7–12 years. The authors further observed that the WHO-based criterion was the least sensitive compared to the IOTF [28], contrary to the present study where the IOTF was least sensitive. Among African children, the evidence is limited. A 2018 pooled analysis of data from a relatively large sample of African school children aged 8–11 years from eight countries reported low sensitivity (29.7%) and high specificity (99.7%) for the WHO BMI definition [11], using deuterium oxide method as reference criterion for body fat [11]. In comparison to the aforementioned African study, the present study reported higher sensitivity (59.4%) but with similar specificity (98.7%). 

The strong positive correlations observed between BMI and percent body fat was similar to the results from previous studies [26,29,30,31]. In a cohort of Swiss school children aged 8–11 years, BMI and body fat were highly correlated particularly in the upper half of the BMI regardless of gender, suggesting that BMI is a good proxy for body fat in children with higher BMI. The authors concluded that BMI could be a good surrogate for body fat in pre-pubertal children [29]. Results from studies that applied body fat derived from deuterium oxide are inconsistent. While the present results echo those among Moroccan adolescents [31], they are contrary to results among Brazilian [32], Australian, and Sri Lankan children [33], where low to moderate associations between body fat and BMI indices were found. Contrary to findings from previous studies [16,17,21], we did not find differences between boys and girls with respect to indices of diagnostic accuracy, although boys tended to have higher values relative to girls.

In comparison to the published BMI cut-offs to diagnose obesity, the optimal cut-offs for the present sample were lower across all criteria. This is not surprising given that the BMI reference cut-offs were generated from diverse populations. The present results suggest that it is appropriate to develop country- and population-specific BMI cut-offs to improve diagnosis of childhood obesity instead of the universal references. For example while the present cut-offs for WHO (0.86 for boys and 0.68 for girls) is similar to that reported in an African sample [11], in an Asian population the corresponding cut-offs were 1.86 for boys and 1.38 for girls [17]. 

These findings have public health implications in the management of childhood obesity. In adults, low to normal BMI with increased body fat is associated with an elevated risk for cardiovascular disease [19,34,35]. The results from the present study indicate that many children with normal BMI-for-age z-scores or percentiles had excess body fat hence BMI could be very useful for detecting excess body fat. Nonetheless, where BMI-for-age is the only criterion in screening children, low sensitivity and moderate sensitivity could lead to misclassification. This is because BMI cannot discriminate body fat and FFM (and the high BMI could be due to high FFM and not necessarily excess fat). This misclassification would lead to missed opportunities for interventions. 

Strengths of the present study are: This is one of the first studies to evaluate the diagnostic performance of the three commonly used international BMI-based reference criteria to detect obesity in sub-Saharan Africa, with data obtained from primary school children in Ghana. The use of deuterium to assess body fat in the children is another strength of this study. The deuterium dilution technique employed in the study is safe, accurate, and non-invasive for assessing body composition and obtaining data on body fat and FFM. There are limitations of the present study that need to be considered in interpretation of the findings. The prohibitive costs of deuterium dilution techniques precluded recruitment of a random-representative large sample with broader age range and generation of body fat percentiles. Additionally, the present results could only be generalized to children aged 8–11 years. There is currently no definite cut-off for body fat with deuterium technique. However, the criteria used [15] is associated with elevated risk of cardiovascular disease and has been consistently used. Moreover, the optimal cut-offs derived in the present study have not been cross-validated in an independent sample.

## 5. Conclusions

Although not significant, the prevalence of obesity varied with the diagnostic criteria applied. BMI z-scores were related to percent body fat in children aged 8–11 years indicating that BMI could be used as a proxy of body fat in this population for screening purposes. The current BMI references for diagnosing obesity in children are largely specific but less sensitive in Ghanaian children. These apparent limitations should be considered by healthcare professionals in diagnosing children. To improve diagnostic accuracy and minimize misclassification, more than one reference could be employed in addition to the direct assessment of body fat and or other health risk factors where practicable. 

## Figures and Tables

**Table 1:** Descriptive characteristics and obesity prevalence of children based on different diagnostic criteria.

Variables	Overall (*N* = 183)	Boys (*N* = 72)	Girls (*N* = 111)	*p*-Value
Median age (y)	10 (9, 10)	10.0 (9, 10)	10.0 (9, 10)	
Median Weight (kg)	30.7 (27.2, 37.5)	29.9 (27.5, 34.9)	31.3 (27.1, 39.1)	0.339
Mean height (cm)	139.5 ± 8.2	138 ± 7.2	140.1 ± 8.81	0.232
Median BMI (kg/m^2^)	15.7 (14.8, 18.0)	15.5 (14.8, 17.1)	16.0 (14.5, 18.7)	0.617
Median BMI z-score	−0.40 (−1.09, 0.63)	−0.51 (−1.08, 0.19)	−0.22 (−1.16, 0.68)	0.387
Median body fat (%)	19.3 (14.1, 26.1)	14.7 (11.6, 21.1)	21.3 (16.7, 27.4)	<0.0001
WHO % (*n*)	11.5 (21)	13.9 (10.0)	9.9 (11)	0.409
CDC, % (*n*)	10.4 (18)	11.1 (8)	9.0 (10)	0.641
IOTF, % (*n*)	8.2 (15)	8.3 (6)	8.1 (9)	0.957
D2O, % (*n*)	17.5 (32)	16.7 (12)	18.0 (20)	0.814

Data are presented as median (25th, 75th percentiles); mean ± SD; percentage (frequency); CDC: Centers for Disease Control and Prevention; D2O: Deuterium oxide; IOTF: International Obesity Taskforce; WHO: World Health Organization; BMI: Body mass index.

**Table 2:** Diagnostic accuracy of BMI-based criteria for defining obesity in children using the percent body fat derived from deuterium method as reference method.

	Sensitivity (95% CI)	Specificity (95% CI)	PPV (95% CI)	NPV (95% CI)
Overall				
WHO	59.4 (40.6–76.3)	98.7 (95.3–99.8)	90.5 (69.6–98.8)	91.9 (86.7–95.7)
CDC	53.1 (34.7–70.9)	99.3 (96.4–99.9)	94.4 (72.7–99.9)	90.9 (72.7–99.9)
IOTF	46.9 (29.1–65.3)	100.0 (97.6–100.0)	100.0 (80.5–100.0)	89.9 (84.3–94.0)
		Boys		
WHO	75.0 (42.8–94.5)	98.3 (91.1–99.9)	90.0 (55.5–99.8)	95.1 (86.5–99.0)
CDC	66.7 (34.9–90.1)	100.0 (94.0–100.0)	100.0 (63.1–100.0)	93.8 (84.8–98.3)
IOTF	50.0 (21.1–78.9)	100.0 (94.0–100.0)	100.0 (54.1–100.0)	90.9 (81.3–96.6)
Girls				
WHO	50.0 (27.2–72.8)	98.9 (94.0–99.9)	90.9 (58.7–99.8)	90.0 (82.4–95.1)
CDC	45.0 (23.1–68.5)	98.9 (94.0–99.9)	90.0 (55.5–99.8)	89.1 (81.3–94.4)
IOTF	45.0 (23.1–68.5)	100.0 (96.0–100.0)	100.0 (66.4–100.0)	89.2 (81.5–94.4)

CDC: Centers for Disease Control and Prevention; IOTF: International Obesity Taskforce; WHO: World Health Organization.

**Table 3:** Optimal cut-point estimation of WHO and CDC criteria for diagnosis of obesity.

	Cut-off	Sensitivity (95% CI)	Specificity (95% CI)	PPV (95% CI)	NPV (95% CI)	AUC (95% CI)
WHO BMI-for-age z-score						
Overall	0.68	93.8 (79.2–99.2)	92.7 (87.3–96.3)	73.2 (57.1–85.8)	98.6 (95.0–99.8)	0.932 (0.885–0.980)
Boys	0.86	91.6 (61.5–99.8)	96.7 (88.5–99.6)	84.6 (54.5–98.1)	98.3 (90.9–99.6)	0.942 (0.857–1.000)
Girls	0.68	95.0 (75.1–99.9)	90.1 (82.1–95.4)	67.9 (47.6–84.1)	98.8 (93.5–99.7)	0.926 (0.868–0.983)
CDC BMI-for-age percentiles						
Overall	69.5	90.6 (75.0–98.0)	90.1 (84.1–94.3)	65.9 (50.1–79.5)	97.8 (93.8–99.5)	0.903 (0.847–0.960)
Boys	87.5	83.3 (51.6–97.9)	91.7 (81.6–97.2)	66.7 (38.4–88.2)	96.5 (87.9–99.6)	0.875 (0.759–0.991)
Girls	69.5	95.0 (75.1–99.9)	89.0 (80.7–94.6)	65.5 (45.7–82.1)	98.8 (93.4–99.9)	0.920 (0.813–0.979)
IOTF BMI-for-age z-score						
Overall	0.50	50.0 (31.9–68.1)	100.0 (97.6–100.0)	100.0 (79.4–100.0)	90.4 (84.9–94.4)	0.750 (0.660–0.840)
Boys	0.50	58.3 (27.7–84.8)	100.0 (94.0–100.0)	100.0 (59.0–100.0)	92.3 (82.5–97.5)	0.792 (0.646–0.937)
Girls	0.50	45.0 (21.1–68.5)	100.0 (96.0–100.0)	100.0 (66.4–100.0)	89.2 (81.5–94.5)	0.725 (0.613–0.837)

CI: Confidence interval; WHO BMI-for-age z-score: World Health Organization Body mass index for age z-score; CDC BMI-for-age: Centers for Disease Control and Prevention; IOTF: International Obesity Taskforce; BMI-for-age: Body mass index-for-age.

## Supplementary Materials

The following are available online at https://www.mdpi.com/1660-4601/17/1/36/s1, Figure S1: Receiver operating characteristics curves for WHO, stratified by gender. Figure S2: Receiver operating characteristics curves for CDC, stratified by gender. Figure S3: Receiver operating characteristics curves for IOTF, stratified by gender.
