# Supplementary material for: Diagnostic Accuracy of Body Mass Index in Defining Childhood Obesity: Analysis of Cross-Sectional Data from Ghanaian Children
Source: Int J Environ Res Public Health. 2019 Dec 19;17(1):36. doi: 10.3390/ijerph17010036 (PMC6981394; doi:10.3390/ijerph17010036)
Supplement: Supplementary file 1 [file ijerph-17-00036-s001.pdf]

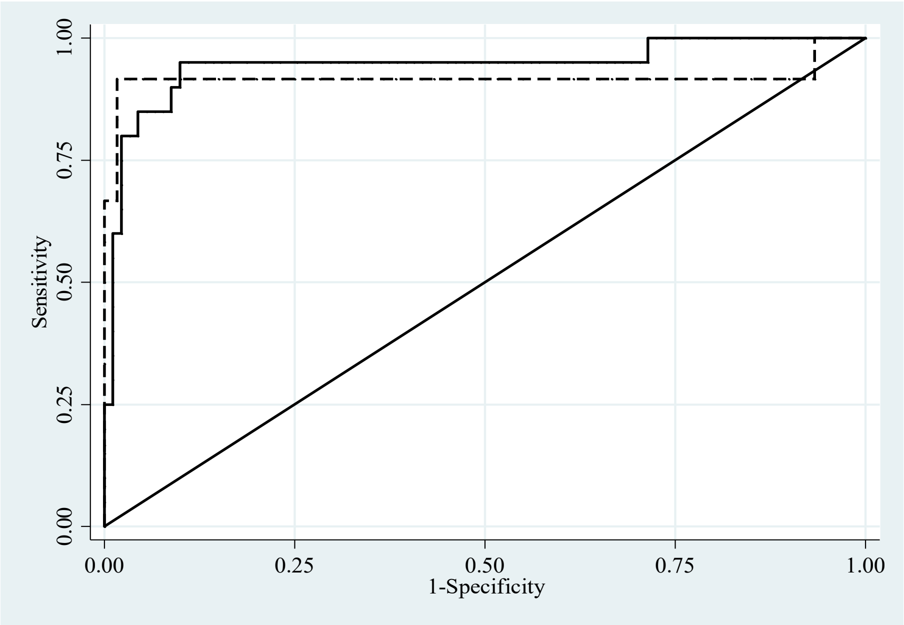

Supplementary Figure S1: Receiver operating characteristics curves for WHO, stratified by gender

Legend: Solid line represents ROC area for girls, AUC = 0.944. Broken line represents ROC area for boys, AUC = 0.918. The diagonal line is the reference line ROC area, AUC = 0.500.

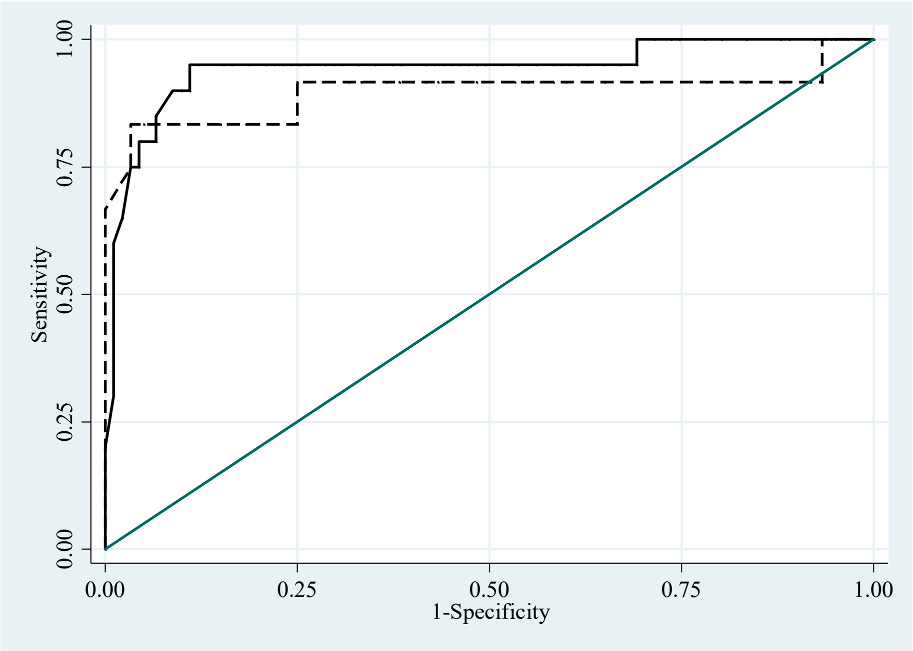

Supplementary Figure S2: Receiver operating characteristics curves for CDC, stratified by gender.

Legend: Solid line represents ROC area for girls, AUC = 0.943. Broken line represents ROC area for boys, AUC = 0.897. The diagonal line is the reference line ROC area, AUC = 0.500.

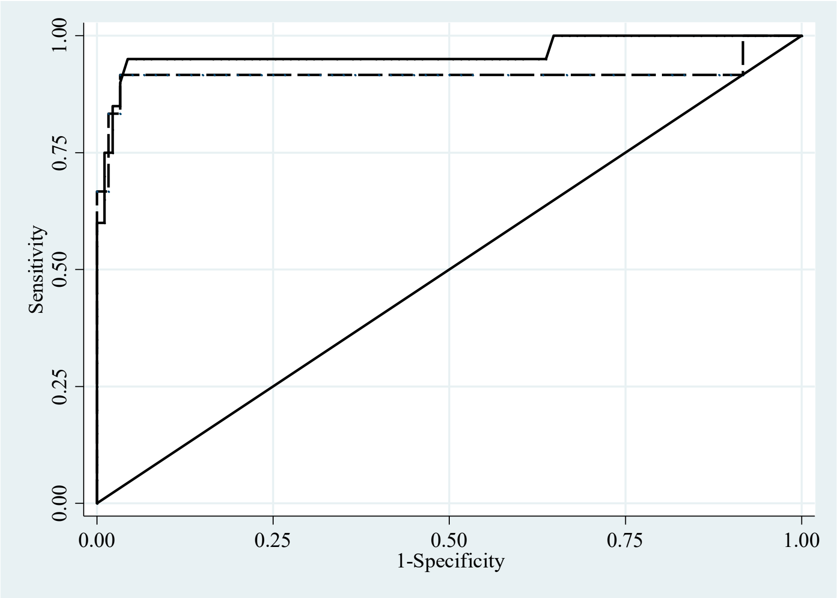

Supplementary Figure S3: Receiver operating characteristics curves for IOTF, stratified by gender.

Legend: Solid line represents ROC area for girls, AUC = 0.960. Broken line represents ROC area for boys, AUC = 0.918. The diagonal line is the reference line ROC area, AUC = 0.500.
